# Dynamic Compression of a SiC Foam

**DOI:** 10.3390/ma15238363

**Published:** 2022-11-24

**Authors:** Eligiusz Postek, Tomasz Sadowski

**Affiliations:** 1 Institute of Fundamental Technological Research, Department of Information and Computational Science, Polish Academy of Sciences, Pawińskiego St. 5B, 02-106 Warsaw, Poland; 2 Faculty of Civil Engineering and Architecture, Department of Solid Mechanics, Lublin University of Technology, Nadbystrzycka St. 40, 20-618 Lublin, Poland

**Keywords:** silicon carbide foam, impact, compression, peridynamics

## Abstract

Silicon carbide foam is a material that can be used as reinforcement of interpenetrated composites. This paper presents an analysis of such a foam subjected to low and fast compression. The analysis is performed using the peridynamics (PD) method. This approach allows for an evaluation of failure modes and such effects of microcracks nucleation, their growth, and, finally, fragmentation. Furthermore, the material appears to behave qualitatively and quantitatively differently while subjected to low- and high-speed steel piston movement. Under slow compression case, damage appears in the entire specimen, but the shape of the structure is not changing significantly, whereas during the fast compression the sample is dynamically fragmented.

## 1. Introduction

The designing process of advanced composites having optimal thermal and mechanical features is strictly related to different phases used for creating novel materials and conditions of the manufacturing process for their production. Different matrix materials are applied for the fabrication of new composites. The most popular are polymers [1,2,3,4,5,6], ceramics [7,8,9,10,11,12,13,14,15,16,17,18,19,20,21], cement [22,23], or metal [24,25,26,27,28,29,30], which joint different kinds of reinforcements, e.g., particles, fibres, and others. The above novel multiphase materials are demanded for space and cars, industrial applications [16], etc. The advanced space systems should ensure the implementation of the scientific and commercial tasks of the missions. Therefore, each composite phase poses different requirements for designing final materials properties, including its architecture. Various internal composite microstructures with different phases contents and reinforcement geometries can be manufactured, from completely disordered structures passing to specially designed architectures in layered, sandwich, or functionally graded materials [31,32,33,34,35], and nanostructures composites [36,37].

A special case of matrix materials can be open-cell foams made of polymer [38,39,40,41,42,43,44], metal [45,46,47,48,49,50], and ceramics [51,52,53,54,55,56,57,58,59,60]. Two types of ceramic foams are important: SiC—silicon carbide foam (SCF) [51,52] and SiOC silicon oxide carbon one [53,54]. Moreover, the rapid progress in nanotechnology leads to the creation of SiC nanostructures in the form of nanowires, nanotubes nanorods [61,62]. Porous SiC nanocrystals are generally produced using two techniques (1) electrochemical etching of bulk materials or (2) embedding SiC crystallites in matrix such as Si [61]. These types of structures exhibit excellence luminescent properties. 

Both mentioned classical foams SFC and SiOC are very good materials for elevated and high-temperature engineering applications, particularly in interpenetrating composites, after filling the foam with the second phase material. The SiC has low thermal conductivity and thermal expansion coefficient [51,52,53,54,55,56]. Various methods are applied for manufacturing of SCFs [55,56,57,58,59,60,63]. They are enabling the fabrication of materials with different levels of open or closed porosity which strongly influence the final macroscopic thermo-mechanical features. Open porosity ceramics are used for various filters in diesel engines, fuel cells, etc. The closed porosity ceramics is applied in case of necessity to get outstanding mechanical strength or thermal insulation.

The internal microstructure architecture is characterised by pore size distribution, orientation, and interconnection. It is related to applied manufacturing technology and strongly influences the mechanical behaviour of the SCFs. The rate of heating during the foaming of SCFs, the ultimate ceramization temperature, and various compositions of starting raw material (including the application of other phases or the existence of impurities) are key factors deciding the final thermo-mechanical characteristics of the SFCs. For example, better thermal insulation requires the SiC to have a higher porosity level. A small amount of porosity in ceramics leads to higher mechanical strength.

Filling the open porosity ceramic foam with metal alloy one can get a relatively new class of materials called interpenetrated phase composite (IPC). This particular case of advanced composite and all others cited above are frequently used for space, military, and car structural parts, which are subjected to extremal loadings during the exploitation like high-velocity dynamic loading, and thermal shocks developing in a very short time interval.

In this paper, we limit theoretical analysis to ceramic foams subjected to compressive loading. The SCFs are typically tested under quasi-static compression (dual ceramic Al_2_O_3_/SiC [57] and single-phase foam Al_2_O_3_ [64] or SiC [65]), using a strain rate of 0.5 mm/min according to ASTM E9 standard. Many factors affect compressive strength, such as open or closed porosity, cell size, strut thickness, and degree of sintering. The strength of the struts has a direct effect on the crushing strength. The foam’s compressive strength increases with the sintering temperature and hence with increased struts strength [57]. However, to the author’s knowledge, there are no papers studying modelling ceramic foam’s behaviour subjected to low, moderate, or higher compressive strain rates. The first attempt to elaborate numerical analysis of the dynamic behaviour of the SCFs subjected to external impact was made in [66], where the foam sample hit the metallic plate with a range of velocities from 15 m/s up to 800 m/s. The highest values of velocities correspond to military applications.

Therefore, in this paper, the numerical analysis of the gradual degradation process of the SiC foam subjected to low, moderate, and high compressive strain rates was modelled starting from the pure elastic response and passing through gradual brittle damage of the struts system, and ending on final fragmentation. The presented analysis demonstrates that the behaviour of the SiC ceramic foam under a low strain rate is qualitatively and quantitatively different in comparison to high strain rate deformation or impact loading [66].

The numerical analysis was performed using the peridynamical approach, which allows for the description of damage initiation, and further degradation growth, leading to fragmentation of the SiC foam. A characteristic feature of the method that starts from crystal mechanics is its non-locality [67,68,69]. In particular, the paper [69] introduces the non-local mechanical field approaches. The paper [70] presents the theory of elasticity in terms of PD. The generalisation of a bond-based model [71] is the state formulation of PD [72]. The latter was applied to materials of brittle behaviour. The monographs [73,74,75] present state-of-the-art of PD. The works [73,74,75,76,77,78] give a broad insight into the examples of the PD description of the behaviour of brittle materials.

The major conclusion resulting from the numerical analysis is that under the high-strain rate loading, the SiC foam load carrying capacity is much higher. Compared to high-impact velocities, damage initiates in high-strain rates much slower.

The next paper will deal with a similar analysis for IPC made of SiC foam filled with AlSi12 alloy.

## 2. Constitutive Model and Formulation

**Figure 1:**
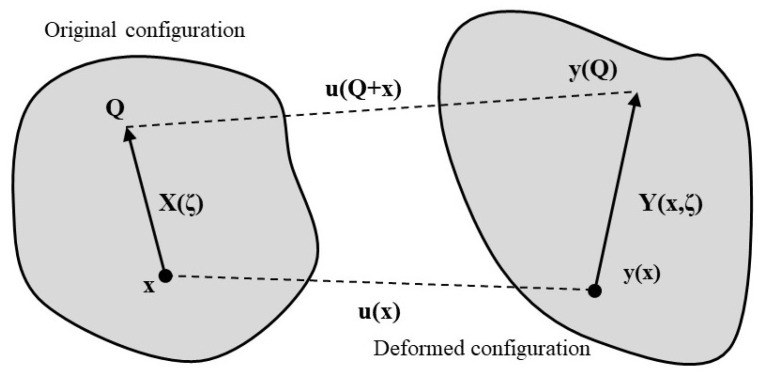
Determination of body deformation.

The ceramic foam is fabricated from the elastic material SiC. The material model depends on peridynamics states, [72], Figure 1. On the left, the original configuration is given, and to evaluate the deformation of the body, two points **Q** and **x** are chosen.

In this non-local model, the deformation is dependent on the change in the distance between positions **Q** and **x** which means bond length **ξ**:

(1) ξ=Q−x

When observing the initial configuration, **X** is a function that acts on the bond **X**(***ξ***). The deformation depends on the new position of the point **x** in the deformed configuration of the body **y**(**x**) and coordinate **Q**, namely, **y**(**Q**):

(2) Y(x,ξ)=y(x+ξ)−y(x)

(3) Y(x,ξ)=y(Q)−y(x)

Now, the displacements are expressed as follows:

(4) U(x,ξ)=u(x+ξ)−u(ξ)

(5) U(x,ξ)=u(Q)−u(x)

The scalar stretch state of the bond e(**Y**) equals:

(6) $$e(Y)=\left|Y\right|-\left|X\right|$$

The scalar stretch state is split up into spherical *e^i^* and deviatoric *e^d^* contributions:

(7) $$e=e^{i}+e^{d}$$

The force state *t*(**Y**) is the sum of its spherical and deviatoric terms:

(8) $$t(Y)=\left(\frac{3k\theta }{m}\right)\omega x+\alpha \omega e^{d}$$

In the formula above, *k*, *θ*, and m are the bulk modulus, dilatation, and weighted volume, respectively. Further on, *ω*, *x*=|**ξ**|, α=15 µ/m are the influence function, scalar state, and a factor dependent on the weighted volume *m* and the shear modulus *μ*, respectively.

**Figure 2:**
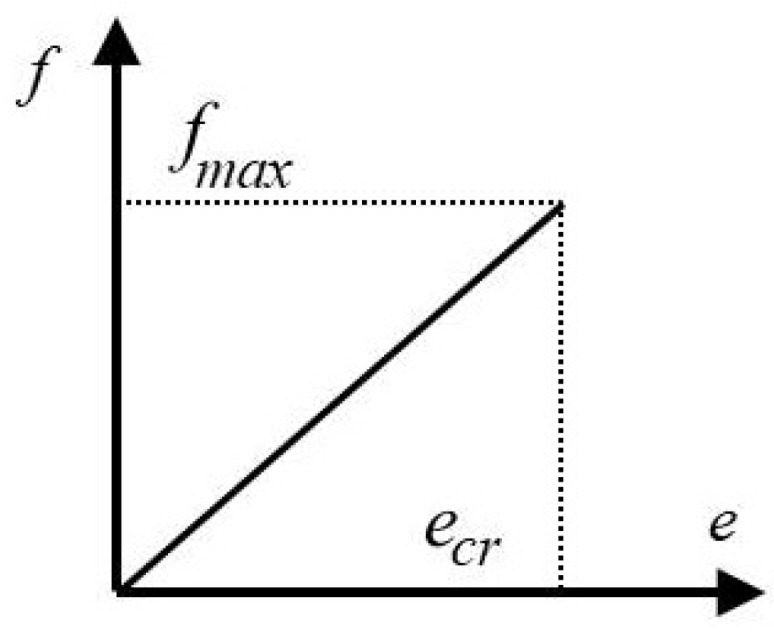
The elastic law and the bond failure condition.

The bond breaks down if the stretch passes the critical threshold, *e_cr_*_,_ Figure 2. The damage is an unreversible phenomenon, and the total damage determines the sum of failed bonds.

The bond-related model [71,72,75] is a particular case of the state-related model. The following relation rules:

(9) f=ceς(x,t,ξ)

where *c* = (18*k*)/(π*h*^4^) is dependent on the horizon *h* and bulk modulus *k*. The force *f* reaches a maximum when the bond stretch is lower than *e_cr_*, *f* = 0 if *e* > *e*_cr_. The function ς equals:

(10) $$\varsigma =\left\{\begin{array}{ccc} 1 & \mathrm{for} & e<e_{cr}\\ 0 & \mathrm{for} & e=e_{cr} \end{array}\right.$$

In this model, progressive degradation of the foam skeleton depends on *G_I_* that is the fracture energy. In our case, it is assumed that the fracture is dominated by mode I. The critical stretch depends on the fracture energy, as follows:

(11) $$e_{cr}=\sqrt{\frac{5G_{cI}}{9kh}}$$

where *G_cI_* is the fracture energy associated with the fracture mode I, *k* is the bulk modulus and *h* is the horizon. The fracture energy is associated with mode I cracking:

(12) $$G_{cI}=\frac{(1-\nu^{2})K_{I}{}^{2}}{2E}$$

In the formula above, the fracture toughness *K_I_* is evaluated experimentally, *E* is Young’s modulus, and *ν* is Poisson’s ratio.

In peridynamics, the damage parameter at a calculation point is defined as:

(13) $$d(x,t)=1-\frac{\int_{\Gamma }\varsigma (x,t,\xi )dv}{\int_{\Gamma }dv}$$

If *d* = 0, the material is pristine, namely, without microcracks. When *d* grows and falls in <0, 1>, the material becomes partially damaged. When *d* = 1, the material is fully damaged.

**Figure 3:**
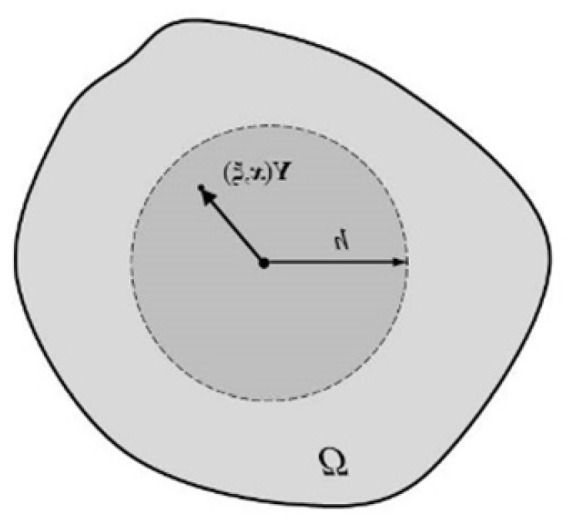
Integration around the calculation point.

The integration at each calculation point is made in the domain *V*, which means a part of the considered body *Ω* surrounding the point with a sphere of the radius *h*, namely the horizon, Figure 3.

## 3. Material Properties and Numerical Model

**Figure 4:**
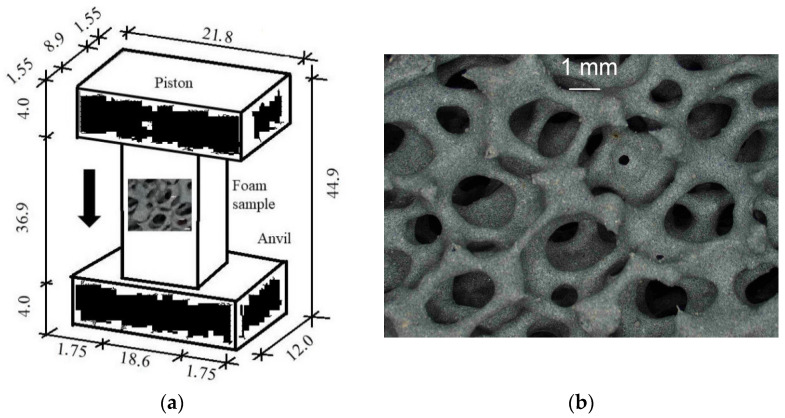
The system under consideration (mm): (**a**) General scheme of the system; (**b**) Structure of the SiC material.

The system under consideration is presented in Figure 4a. It consists of an anvil, a foam sample, and a piston. The piece is 34.7 mm in height, 8.9 mm thick, and 18.6 mm in width. A piston compresses the sample against an anvil with constant velocity *V*.

The internal structure of the sample is obtained using micro-CT scanning with Sky Scan 1174 (Bruker) apparatus [79]. A fragment of the microscopic image of the material is shown in Figure 4b. First, the MIMICS program [80] is applied to reconstruct the geometry of the sample and receive the initial structured tetrahedral discretization. Then, the GMSH program [81,82] is applied to convert the initial mesh into the unstructured mesh. Before entering the GMSH, the outer triangularized surface of the initial 3D mesh is taken using the GiD [83] program. Then, the surface is smoothed using MSC Patran [84]. Finally, the 3D tetrahedral mesh is obtained with the GMSH.

**Figure 5:**
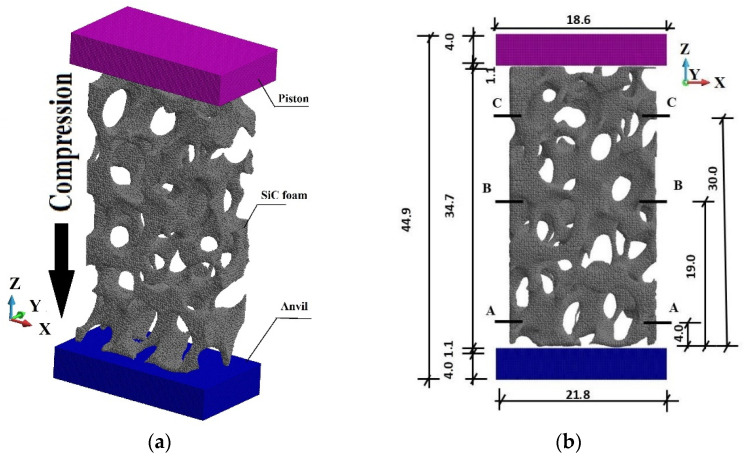
The PD discretized system, SiC foam placed between piston and anvil (mm): (**a**) Axonometric view; (**b**) Plane view.

**Figure 6:**
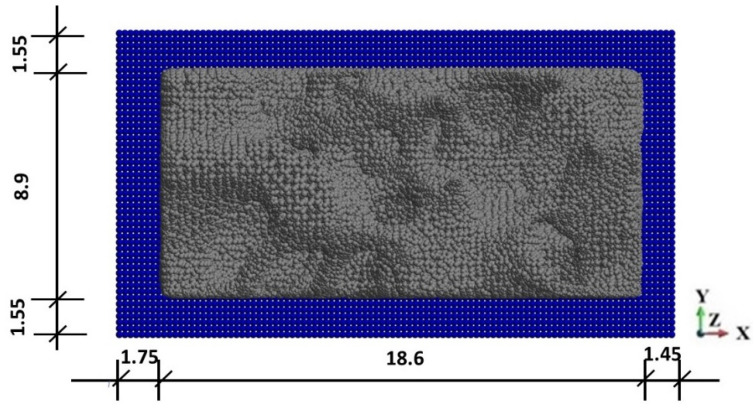
The PD discretized view from atop, without piston (mm).

The SiC sample is placed between the piston and the anvil, Figure 5 and Figure 6. The contact conditions are assumed between the sample, piston, and anvil. The penalty formulation with the penalty number 1.0 × 10^12^ is used. The friction coefficient is taken as 0.3. Since the shape of the foam is complex with irregular branches and openings, general contact conditions, including self-contact, are applied.

The SiC sample discretization counts 261,496 calculation points. The anvil and the piston are discretized with 100,000 calculation points each. The horizon value for the anvil and the piston is assumed to be 30.0 × 10^−04^ m, and for the SiC sample is 6.5 × 10^−04^ m. The dimensions of the horizon fulfil the requirement of their minimum size, which is three times bigger than the maximum distance between the calculation points. The criterion has been evaluated in [85].

The material properties of the SiC sample are as follows: the elastic properties of the foam, namely, Young’s modulus is 430.0 GPa, Poisson’s ratio is 0.37, and mass density is 3200 kg/m^3^. In addition, the fracture toughness is 4.1 MPa.m^1/2^ [86]. Therefore, the calculated critical value for the SiC sample is *e_cr_* = 1.0646 × 10^−05^. The piston and the anvil are fabricated of steel of Young’s modulus 210 GPa, Poisson’s ratio 0.3, and mass density 7850 kg/m^3^.

The sample is subjected to fast compression with the velocity of the piston ranging from *V* = 40 m/s up to 440 m/s. The calculations are made with the dynamic explicit solver of the system Peridigm [85,87]. The program is reliable since it has been verified with many examples so far [76,78,88]. The time of the analysis is 3.06 × 10^−05^ s. The stable time step is 3.06 × 10^−08^ s. During the integration, the constant time increment of 2.0 × 10^−08^ s is used. The applied time increment is well below the stable time step. The program is implemented on the high-performance Cray XC60 Linux cluster, where the calculations were done. The production run required 4100 s using 1920 cores.

**Figure 7:**
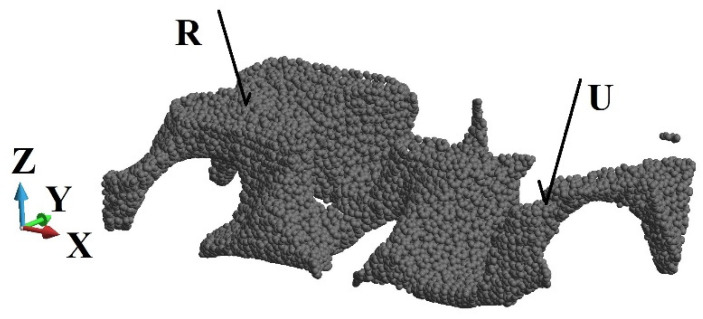
Observed points at cross-section A-A (Figure 5b).

**Figure 8:**
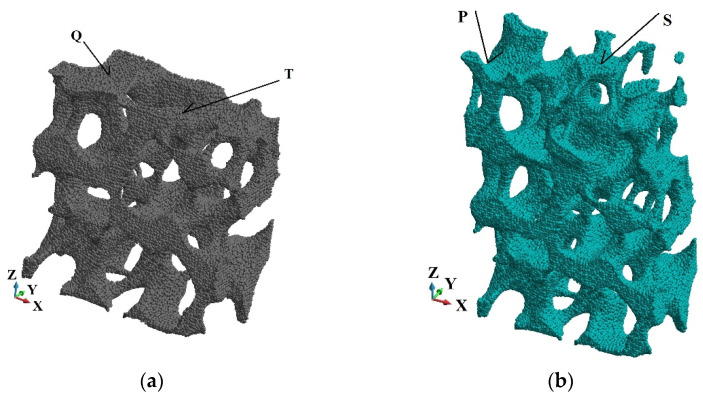
The observed points: (**a**) Cross-section A-A; (**b**) Cross-section B-B.

In the course of the analysis, the damage variable is followed at the six points in the three cross-sections, A-A, B-B, and C-C, as indicated in Figure 5b. The observed points are shown in Figure 7 and Figure 8.

## 4. Numerical Results

The piston presses the SiC foam sample from the top, Figure 5a. While compressed, the sample undergoes damage and, in the case of high-velocity compression, fragmentation. The foam was subjected to compression with the piston velocities of the range *V* = 40 m/s to 440 m/s. There are chosen three velocities of the piston to present the results, namely *V* = 40 m/s, 240 m/s, and 440 m/s.

**Figure 9:**
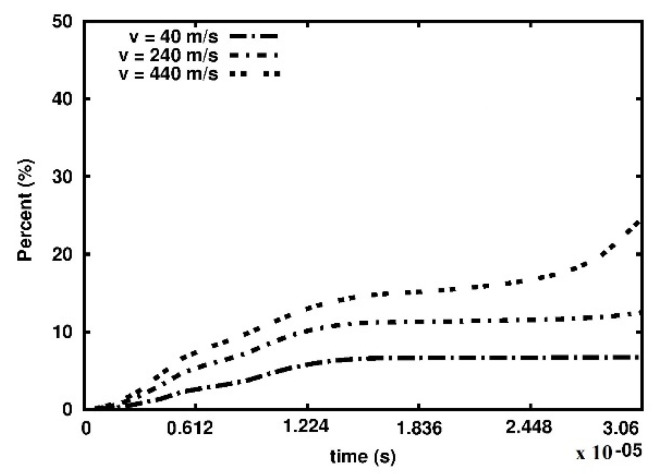
Percent of the damaged volume of the sample in time.

In Figure 9 the total damage of the foam versus time plots is presented. It has been arbitrarily assumed that the volume of the material surrounding the calculation point is fully damaged when the *d* parameter is higher than 0.95. For low- and medium-velocity of the piston, the damage growth stabilizes approximately at 1.22 × 10^−05^ s. In the high-velocity case, three growth stages can be recognized, namely,

(1) damage growth;
(2) damage stabilization;
(3) fast damage growth.

For this case, the fast damage growth phase starts close to the end of the process at about 2.7 × 10^−05^ s.

**Figure 10:**
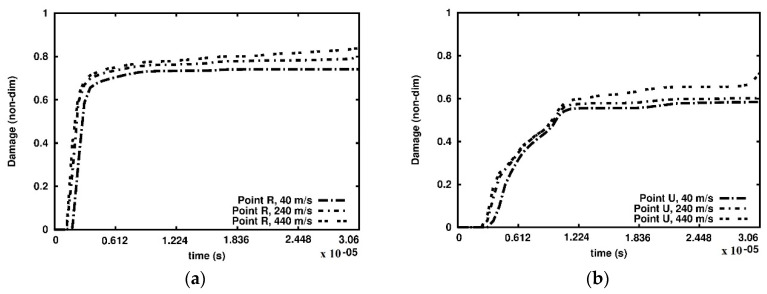
Damage parameter variation, cross-section A-A: (**a**) point R; (**b**) point U.

**Figure 11:**
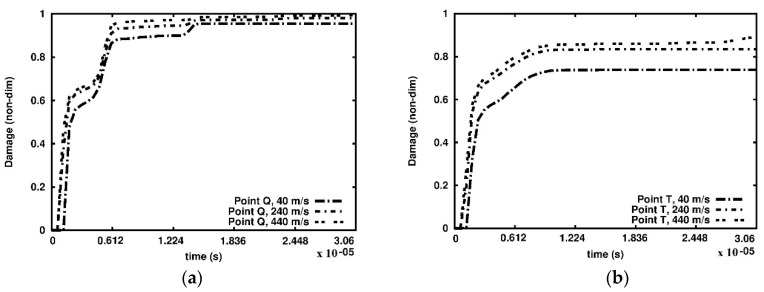
Damage parameter variation, cross-section B-B: (**a**) point Q; (**b**) point T.

**Figure 12:**
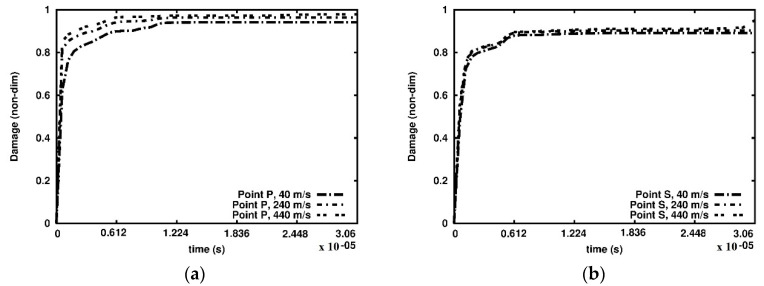
Damage parameter variation, cross-section C-C: (**a**) point P; (**b**) point S.

Furthermore, we analysed the damage growth in three cross-sections at selected points as indicated in Figure 7 and Figure 8. The damage versus time plots are given in the cross-sections A-A, B-B and C-C in Figure 10, Figure 11 and Figure 12, respectively. Due to the foam’s complex and irregular shape, the selection of the points is quite arbitrary. Therefore, the observed curves allow for quantitative conclusions rather than qualitative ones only. The damage variable is observed at the time interval 0.6 × 10^−06^ s. In general, the damage is the lowest at the points R and U of the cross-section A-A closest to the anvil, Figure 10. The damage appears at points P and S of the cross-section C-C near the piston almost immediately, Figure 12, which contrasts the cross-sections B-B, Figure 11, and A-A, where a delay occurs. The biggest delay in damage presence is at point U located in the cross-section A-A. The time instances at which the damage appears, and the damage values are collected in Table 1, Table 2 and Table 3.

**Figure 13:**
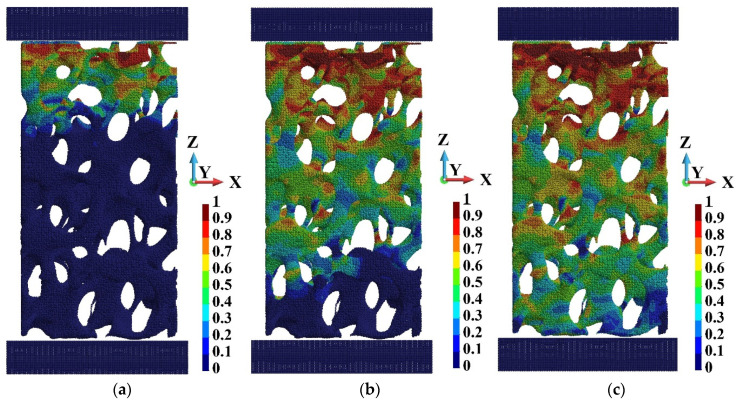
Damage advancement for the piston velocity 40 m/s at time instants: (**a**) 0.6 × 10^−06^ s; (**b**) 0.3 × 10^−05^ s; (**c**) 0.42 × 10^−05^ s.

**Figure 14:**
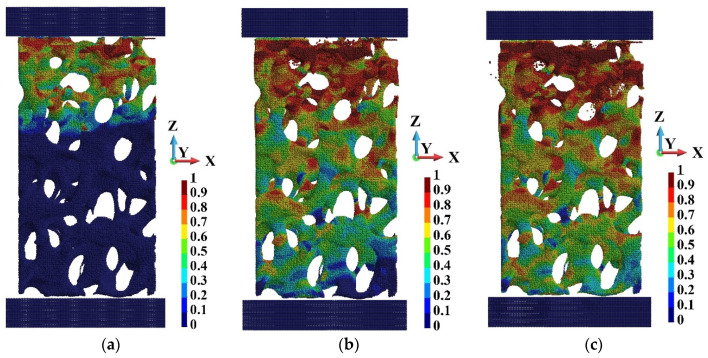
Damage advancement for the piston velocity 440 m/s at time instants: (**a**) 0.6 × 10^−06^ s; (**b**) 0.3 × 10^−05^ s; (**c**) 0.42 × 10^−05^ s.

Figure 13 and Figure 14 present damage development along the sample’s height at the beginning of the process. The last instant, namely, 0.42 × 10^−05^ s, is chosen when damage appears in the entire sample compressed with the highest velocity. The damage advances faster in the case of high-velocity compression, which is visible in Figure 13b and Figure 14b, the most distinctly. When the sample undergoes high-velocity compression, the damage appears almost in the whole sample at time 0.42 × 10^−05^ s. However, in the case of low-velocity compression, the undamaged regions still exist, Figure 13c and Figure 14c.

**Figure 15:**
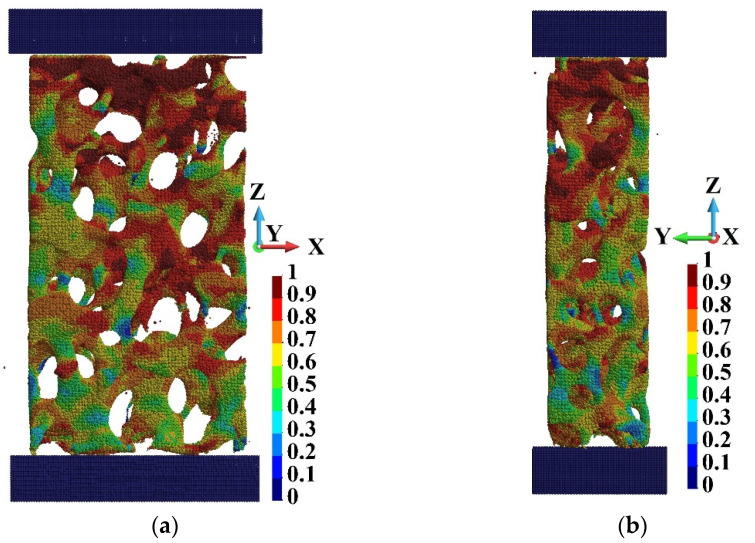
Damage distribution and shape of the foam under piston velocity 40 m/s at the end of the process: (**a**) X-Z plane view; (**b**) Y-Z view.

**Figure 16:**
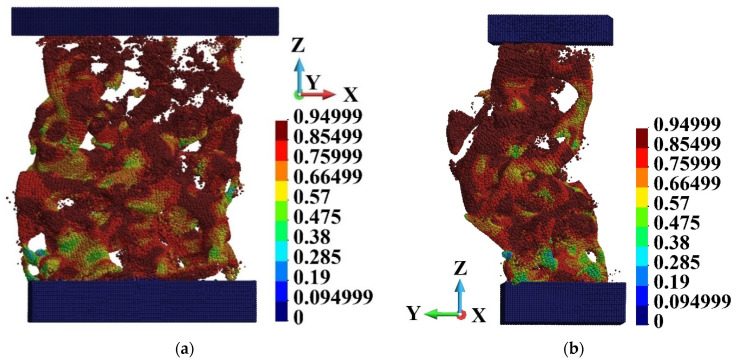
Damage distribution and shape of the foam under piston velocity 440 m/s at the end of the process: (**a**) X-Z plane view; (**b**) Y-Z view.

A significant difference exists between the state of the sample when subjected to the low-velocity action of the piston and the high-velocity action, Figure 15 and Figure 16, respectively. In the case of low-velocity motion of the piston, the shape of the foam sample remains similar to the original one. In contrast, at the high-velocity compression when the sample deforms one can observe fragmentation at the end of the degradation process. The out-of-plane displacement appears late in the process and is sudden, resembling buckling. It corresponds with the fast growth of damage as well.

**Figure 17:**
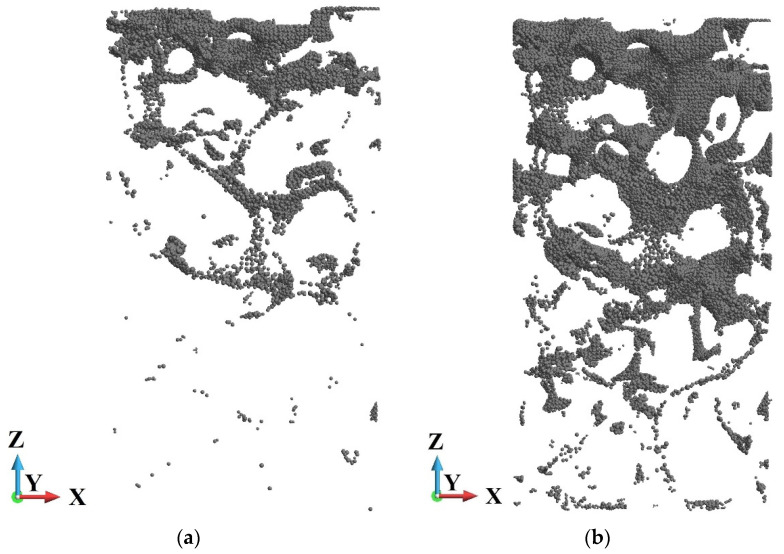
Points at which damage variable is higher than 0.95 at the end of the process: (**a**) piston velocity *V* = 40 m/s; (**b**) piston velocity *V* = 440 m/s.

A comparison of the damage distribution for the cases of low- and high-velocity compression is shown in Figure 17. There are shown points at which damage variable *d* is higher than 0.95. The damage is more advanced closer to the piston than the anvil side in both cases. A characteristic feature of damage distribution is the creation of “chains” of damaged points in the foam branches where the damage starts to develop.

**Figure 18:**
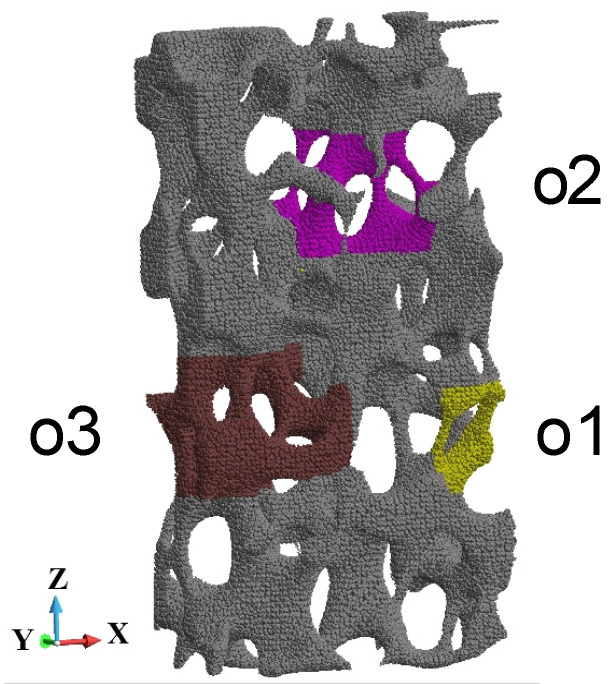
Regions of the sample chosen for detailed analysis, region o1 in yellow, region o2 in pink, and region o3 in brown.

**Figure 19:**
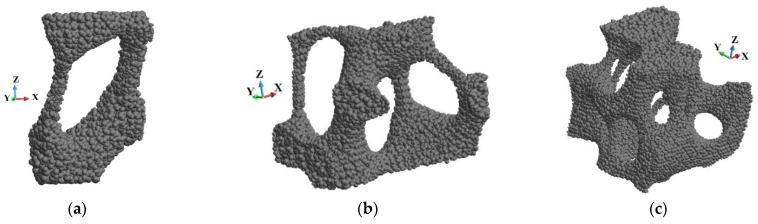
The enhanced details of the foam microstructures: (**a**) region o1; (**b**) region o2; (**c**) region o3.

The further analysis concerns detailed observations of the failure of the foam. It has been chosen three regions arbitrarily, namely o1, o2, and o3, Figure 18. The regions are presented in detail in Figure 19. They are slightly rotated to enhance the small rods that constitute the foam cells. The selected rods are of interest since they are crushing during the loading process. We focused our attention on the end of the analysed impact process with the piston velocity 440 m/s. This is because the sample becomes fragmented during failure.

The cells of the foam are opened, Figure 19. It is due to the fabrication process and technological requirements. A fluid or fluid-like metal can easily penetrate the sample. The structure of each cell contains several elements that can be considered thin rods. The rods are destroyed first during the impact process, which is presented further.

**Figure 20:**
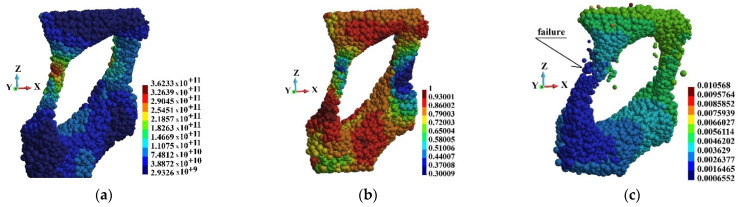
Region o1: (**a**) Mises stress distribution (Pa); (**b**) damage distribution; (**c**) displacements (m) in deformed configuration, scale factor 0.2.

**Figure 21:**
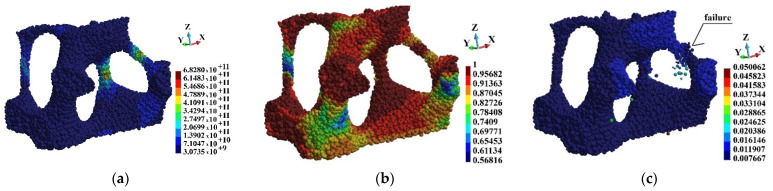
Region o2: (**a**) Mises stress distribution (Pa); (**b**) damage distribution; (**c**) displacements (m) in deformed configuration, scale factor 0.2.

**Figure 22:**
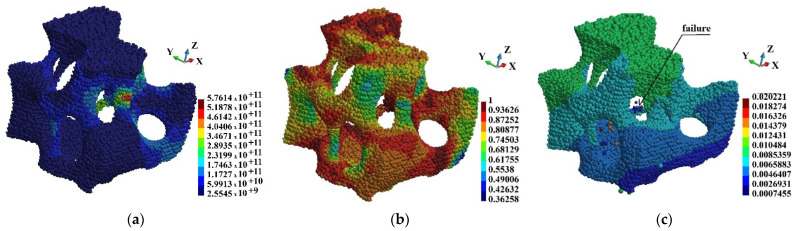
Region o3: (**a**) Mises stress distribution (Pa); (**b**) damage distribution; (**c**) displacements (m) in deformed configuration, scale factor 0.2.

Figure 20, Figure 21 and Figure 22 present Mises stress, damage parameter distribution and displacement field in the selected regions. The displacement field is shown in the deformed configuration. The latter allows for presentation of the failure mechanism of the cells. Therefore, hints concerning the failure of the entire foam can be obtained. When observing Figure 21 and Figure 22, it has been found that the rods fail first. The failure takes place in the regions where Mises stress concentrates and the damage parameter is high. The thin rods failure appears in each of the regions that are observed. Therefore, it can be concluded that the thin rods failure happens in almost entire foam. In consequence, it is the mechanism of failure of entire brittle structure.

## 5. Conclusions

In this paper, the analysis of fast and slow compression of a SiC porous sample applying the peridynamics method is presented. This approach allows for the evaluation of failure modes and such effects as microcracks nucleation and growth, and finally fragmentation. The investigated system for dynamic compression (Figure 4a) consists of an anvil, a foam sample, and a piston. The piston compresses the sample against an anvil with different velocities *V* = 40 m/s, 240 m/s, and 440 m/s.

It has been observed qualitatively different behaviour and failure of the specimen depending on the speed of the piston. The main conclusions are as follows:

- When the specimen undergoes fast compression, contact between the branches appears;
- In the slow compression case, damage appears in the entire specimen, but the shape of the structure is not changing significantly, which means the specimen undergoes failure due to microcracks;
- In the fast compression conditions, the specimen is fragmented, and the displacements become large and out of the plane; The final shape resembles buckled structure;
- In the observed time interval, the strongly damaged volume (d > 0.95) stabilizes in time for low and medium piston velocities, but for high piston velocity, the damaged volume starts to grow fast at the end of the interval. It corresponds to fragmentation phenomenon in this case;
- Failure of the thin rods in the open cells happens in almost the entire structure of the foam;
- Failure of the thin rods is the main reason for the change in the foam configuration and the foam fragmentation due to loss of continuity between the fragments of the structure.

The porous SiC material serves as a skeleton of the alumina-infiltrated composite. Further research will focus on the dynamic behaviour of such a composite.

## Figures and Tables

**Table 1:** Time instance (s) when damage appears at observed points and the damage value, cross-section A-A.

Cross-Section	A-A			
Point	R		U	
Velocity	*t* (s)	*d*	*t* (s)	*d*
40 m/s	0.18 × 10^−05^	0.10 × 10^−01^	0.36 × 10^−05^	0.30 × 10^−01^
240 m/s	0.18 × 10^−05^	0.26	0.30 × 10^−05^	0.32 × 10^−01^
440 m/s	0.18 × 10^−05^	0.40	0.3 × 10^−05^	0.58 × 10^−01^

**Table 2:** Time instance (s) when damage appears at observed points and the damage value, cross-section B-B.

Cross-Section	B-B			
Point	Q		T	
Velocity	*t* (s)	*d*	*t* (s)	*d*
40 m/s	0.18 × 10^−05^	0.48	0.12 × 10^−05^	0.16 x 10^−02^
240 m/s	0.12 × 10^−05^	0.37	0.12 × 10^−05^	0.19
440 m/s	0.12 × 10^−05^	0.47	0.12 × 10^−05^	0.27

**Table 3:** Time instance (s) when damage appears at observed points and the damage value, cross-section C-C.

Cross-Section	C-C			
Point	P		S	
Velocity	*t* (s)	*d*	*t* (s)	*d*
40 m/s	0.6 × 10^−06^	0.62	0.6 × 10^−06^	0.48
240 m/s	0.6 × 10^−06^	0.81	0.6 × 10^−06^	0.55
440 m/s	0.6 × 10^−06^	0.85	0.6 × 10^−06^	0.57

## Data Availability

The data is not available due to ongoing project.
